# Design of Citrus Fruit Detection System Based on Mobile Platform and Edge Computer Device

**DOI:** 10.3390/s22010059

**Published:** 2021-12-23

**Authors:** Heqing Huang, Tongbin Huang, Zhen Li, Shilei Lyu, Tao Hong

**Affiliations:** 1 College of Electronic Engineering (College of Artificial Intelligence), South China Agricultural University, Guangzhou 510642, China; huangheqing@stu.scau.edu.cn (H.H.); 20203163156@stu.scau.edu.cn (T.H.); lvshilei@scau.edu.cn (S.L.); 2 National Research Laboratory of Mechanization of Citrus Industry Technical System, Guangzhou 510642, China; 3 Guangdong Agricultural Information Monitoring Engineering and Technology Research Center, Guangzhou 510642, China; 4 Pazhou Lab, Guangzhou 510330, China; 5 School of Electronic and Information Engineering, Beihang University, Beijing 100190, China; hongtao@buaa.edu.cn

**Keywords:** citrus detection, UAV, mobile operation platforms, edge computing devices

## Abstract

Citrus fruit detection can provide technical support for fine management and yield determination of citrus orchards. Accurate detection of citrus fruits in mountain orchards is challenging because of leaf occlusion and citrus fruit mutual occlusion of different fruits. This paper presents a citrus detection task that combines UAV data collection, AI embedded device, and target detection algorithm. The system used a small unmanned aerial vehicle equipped with a camera to take full-scale pictures of citrus trees; at the same time, we extended the state-of-the-art model target detection algorithm, added the attention mechanism and adaptive fusion feature method, improved the model’s performance; to facilitate the deployment of the model, we used the pruning method to reduce the amount of model calculation and parameters. The improved target detection algorithm is ported to the edge computing end to detect the data collected by the unmanned aerial vehicle. The experiment was performed on the self-made citrus dataset, the detection accuracy was 93.32%, and the processing speed at the edge computing device was 180 ms/frame. This method is suitable for citrus detection tasks in the mountainous orchard environment, and it can help fruit growers to estimate their yield.

## 1. Introduction

Citrus is the largest fruit in the world and one of the main cash crops. With the gradual increase of citrus yield every year, the planting range is also gradually expanded, people pay more attention to this highly nutritious fruit. In this case, fruit farmers’ yield increase, and yield estimation play a role in economic income generation. Target detection algorithm can provide technical support for these citrus tasks. In recent years, it has been widely used in citrus operations [1], such as citrus picking [2], orchard yield measurement [3]. At present, the target detection network built by the deep and wide convolutional neural network has ideal recognition accuracy and real-time performance. Still, many deep and wide target detection algorithms need to process on the server with high configuration. A large amount of memory consumption and existing occupation make running on the edge computing platform; on the other hand, in the actual mountain orchard environment, with undulating terrain, complex terrain, and different soil thickness, it is dangerously challenging to collect citrus data. At the same time, the data taken by the regular use of the mobile camera is relatively single, which is greatly affected by the site, thus affecting the judgment of fruit farmers on the estimation of citrus yield. Therefore, it is of great significance to study the acquisition and transmission of citrus images on the mobile operating platform/UAV and realize the accurate recognition of citrus targets for the fine management and yield estimation of orchards.

The traditional machine learning method [4,5] is not ideal for target recognition, and neither speed nor precision can meet the actual needs. In 2012, the proposal of convolutional neural network [6] promoted the upsurge of deep learning, and many target detection models [7] were proposed. At present, target detection algorithms can divide into two categories. One is based on anchor-free type; the main methods are FCOS [8], Centernet [9], etc., instead of setting anchor boxes artificially, objects are detected by point regression. The other is based on anchor-based, which is more used, such as YOLO [10,11], SSD [12], etc., by setting anchor boxes with different sizes and proportions for each pixel. Specifically, the anchor-based target detection algorithm takes the anchor as the detection starting point, corrects the position and category of the anchor, and obtains the results; this kind of algorithm needs to manually set more parameters, which can more effectively get the prior knowledge of the data set and make the model converge faster. The anchor-free target detection algorithm uses anchor points and key points to pair the objects to be detected; this method reduces the parameters, simplifies the calculation process of the algorithm, and can achieve good results in speed and accuracy.

In the detection task in the agricultural field, SA et al. [13] used the transfer learning method to fine-tune the weight of pre-training and detect the fruit, this method takes into account the detection accuracy and recall, but the model training time is long; Tian et al. [14] used the improved YOLOv3 detection algorithm to process high-resolution image data in real-time, which can quickly detect fruits in orchards; Chen et al. [15] proposed an unsupervised learning method, which uses the target detection algorithm in deep learning to learn plane features, which solves the problem of slow traditional recognition and matching methods; Kurtulmus et al. [16] extracted and fused multi-scale features from citrus images to obtain high detection accuracy, but added multi-stage complex search strategy will reduce the detection speed of the model.

With the update of hardware devices, many high-performance sensors are widely used [17,18]. In some specific scenarios, these low-power and portable devices may play an important role, to obtain crop information accurately and timely. Fan et al. [19] used UAV low altitude remote sensing technology to obtain crop images with different resolutions, summarized the research and application of crop growth trend, yield estimation, pest, and forage monitoring, and achieved good results; Liu et al. [20] proposed to transform the model based on color space, segment the foreground (crop) and background (soil background) of UAV image, obtain the classified binary image of the image, and then extract the plant number information of corn seedling image by skeleton extraction algorithm. Some studies have deployed target detection algorithms to edge computing devices to complete different tasks [21,22,23]. In orchard scenarios, accurate real-time detection or classification goals effectively judge crop health and yield-related management. Mazzia et al. [24] implemented the YOLOv3-Tiny target detection algorithm on different embedded platforms. The proposed edge segment solution can deploy on unmanned ground vehicles, and the apples in the orchard can be detected and counted in real-time with a detection accuracy of 83.64%. SA et al. [25] trained six models by adjusting the number of channels in different depths and widths of the target detection model to detect weeds on Jetson TX2 to reduce damage to surrounding plants.

We presented a citrus fruit recognition method based on a deep learning target detection model, mobile data platform, and edge computing device. Specifically, we place an unmanned aerial vehicle in the orchard to collect citrus images from all directions around the citrus tree. These data are transmitted to the edge computing device Jetson nano via 4G data transfer. An improved target detection algorithm is deployed inside the device. To solve the problem that it is difficult to balance the accuracy and real-time of target detection algorithm in edge computing device, the state-of-the-art model was improved the structure of the model by adding a convolution module (CBAM) [26] with an attention mechanism; fusion of feature maps with different resolutions using adaptive parameters [27]; finally, with the accuracy guaranteed, we prune the model weights, add L2 regularization constraints to the batch normalization layer, delete and fine-tune the 30% redundant channels that occupy the minor information in the channels. In the experimental part, the feasibility of the proposed method proves by ablation experiments of the model itself and comparison experiments with other models.

The main contributions of this paper are as follows:

(1) Combined with the advantages of the mobile operating platform and edge computing equipment, the improved deep learning target detection model is used to accurately and real-time detect the omni-directional citrus fruit image taken by UAV.
(2) The target detection algorithm was improved and optimized. Attention mechanism, multi-layer feature adaptive fusion, and pruning optimization are adopted to improve the accuracy and reasoning speed of the model.

## 2. Data Acquisition and System Design

### 2.1. Collection and Transmission of Citrus Fruit Data Set

**Figure 1:**
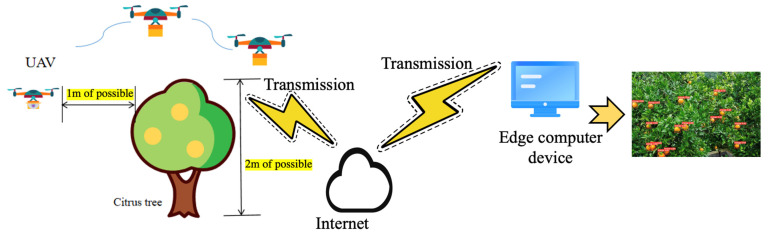
UAV device for taking citrus images. The citrus tree is about 2 m high. We used UAV to collect images in an all-round way at about 1 m around the fruit tree.

The scene of UAV shooting citrus fruit image is shown in Figure 1. To obtain citrus images in a mountain orchard environment, on the one hand, we need some necessary hardware equipment, such as a camera, power supply, visual display screen, and edge computing equipment, to recognize citrus images. On the other hand, while obtaining the above information, the rational use of UAV technology can help fruit farmers monitor citrus fruits in an all-around way, improve orchard management efficiency, and assist in yield estimation. The UAV equipment used in this paper is Mavic air 2, it is produced by Dajiang and released on 28 April 2020, which can take high-pixel photos and fly at low altitudes for one hour. The main task of the UAV is to collect image data about 1 m above the citrus fruit tree and upload the image to the edge computer device. The image is further processed by using the target detection algorithm to achieve the real-time detection effect of citrus fruit.

**Figure 2:**
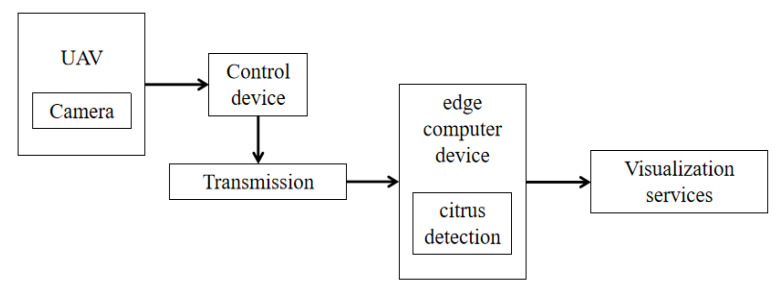
Citrus image shooting and transmission process.

UAV capture image has high security and flexibility. The change of environment has little impact on its image acquisition, and the image acquisition cycle is also short, which improves work efficiency and reduces the labor cost. Figure 2 depicts the process of capturing citrus images using UAV. First, we used the camera carried on the UAV to capture the citrus image, transmit it to the edge computing device through the wireless network. Then, we used the deployed target detection algorithm to detect the citrus and visualize it through the display device.

We used the self-made citrus data set to train the model provided in this paper and collected data in the citrus orchard in Yizhang County, Hunan Province, China. A total of 1800 citrus fruit images taken by mobile camera and UAV low altitude aerial photography, about 1 m away from citrus fruit trees, and annotated manually with LabelImg. The dataset system is used as a format for coco datasets to standardize training data. We selected 400 aerial images as the test set, 1300 citrus images as the training set, and 100 as the verification set. We note that the problem of leaf occlusion and citrus fruit occlusion significantly impacts the recognition effect. The test set is manually divided into two parts: A and B. A test set contains 250 slightly occluded citrus fruit images, and the B test set includes 150 severely occluded citrus fruit images.

### 2.2. Data Enhancement

To strengthen the richness of training samples’ richness and increase model robustness, three data enhancement methods are used in this paper, namely Mixup, Cutout, and Cutmix. Mixup randomly selects two images from the training samples and mixes them in proportion but does not affect the labeling results. Cutout randomly cuts out a particular area in the chosen image and fills in a specific pixel value, which does not affect the labeled result. Cutmix randomly cuts the samples in the training set, but does not fill in fixed pixel values and uses another randomly selected sample area to fill in the image quality. In the citrus dataset, there are severe problems of mutual occlusion and leaf occlusion between citrus. Through data enhancement, the model is more inclined to recognize objects from local images, enhance the model’s positioning ability, and improve the robustness.

### 2.3. Design of Edge Computing Device

**Figure 3:**
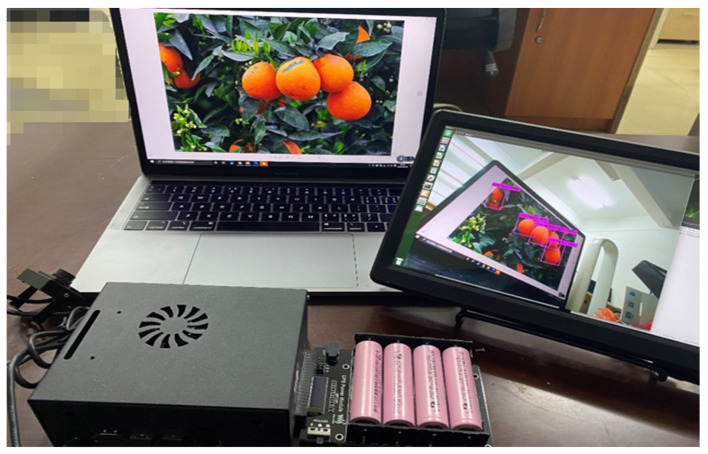
Portable edge computing device with a small visual display, mobile power supply, camera, Jetson nano, and internal target detection model.

The hardware equipment of the citrus fruit recognition system is mainly composed of an edge computing development board, display screen required for visualization, external camera, and power supply module. We deployed the target detection model to Jetson nano for reasoning, which is a portable development version of artificial intelligence released by NVIDIA. It contains 128 core Maxwell architecture GPU and fast reasoning speed in 5 W/10 W low-power model, has rich AI programming neurons, and speeds up the speed of deep learning algorithm for equipment operation. The operating system is Ubuntu 18.04. The platform power supply module is 5000 mAh, 12 V equalizing rechargeable lithium battery; powerful computing power and small platform design are conducive to the design of portable citrus fruit target detection system. Figure 3 shows an example diagram of an edge computing device for detecting citrus fruits.

## 3. Construction of Citrus Target Recognition Model

### 3.1. Basic Model Selection

YOLOv5 is a one-stage target detection algorithm that combines detection speed and detection accuracy. The detection network has four versions, in turn, YOLOv5x, YOLOv5l, YOLOv5m, YOLOv5s. Among them, YOLOv5s is the network with the minor depth and width of the feature map, and the other three can be based on it, which has been deepened and widened.

**Figure 4:**
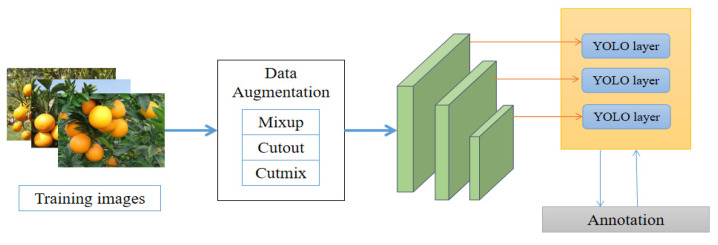
The YOLOv5 model structure diagram. We used three data enhancement methods during training.

Considering the accuracy and real-time of the citrus fruit detection model in edge computing device deployment, we used YOLO5s with minor parameters and the fastest speed as the benchmark of the detection model. Structurally, the model first increased the Focus structure; the core of this structure is to slice pictures, change the size of the feature map of the input image, use many residual CSP structures, enhance the learning ability of the model; add a variety of data enhancement operations. In the training process, the drop block is used to prevent overfitting. As for the drop block method, it is suitable for the regularization of the convolution layer, and the adjacent areas on the feature map of a layer are deleted together, rather than independent pixels. Therefore, the model will pay more attention to other places to fit the data. The loss function used the CIoU border loss function constraint model, the overall architecture shown in Figure 4.

### 3.2. Module Design of Attention Mechanism

**Figure 5:**
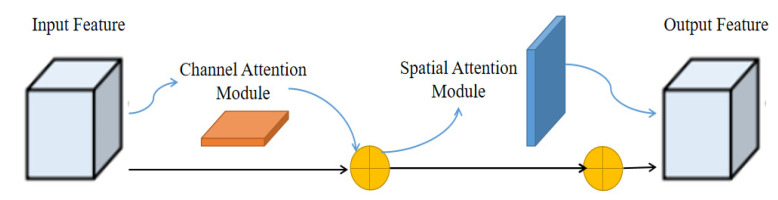
CBAM architecture is shown in the figure, which is composed of two serial modules: channel attention and spatial attention.

When detecting the images captured by UAV low altitude aerial photography, due to the difficulty in grasping the flight height and the uncertain scale transformation of the collected data, which is easy to cause the problem of significant differences of citrus size in different images, at the same time, there are some problems between different citrus, such as mutual occlusion and leaf coverage, which makes it a great challenge to detect such data. This paper used a lightweight attention mechanism module CBAM to strengthen attention to occluded objects. This architecture is inserted into many CNN models without disturbing the model training steps. Through the feature map output by the model, the attention mechanism carries out in channel and space simultaneously. The architecture diagram of CBAM is shown in Figure 5.

Among them, the formula for calculating channel attention is shown in Equation (1). The input characteristic map is obtained by global pooling operation and average pooling operation of width and height, respectively. Then, they are sent to a two-layer neural network to share parameters at the same time. After fusion, they activate by sigmoid activation function to generate the characteristic map required for channel attention.

(1) $$M_{c}\left(F\right)=\sigma (\mathrm{MLP}\left(\mathrm{Avgpool}\left(F\right)\right)+\mathrm{MLP}(\mathrm{Maxpool}(F)))$$

The formula for calculating spatial attention is shown in Equation (2). The feature mapping and original mapping of computational channel attention fusion are used to calculate spatial attention. Perform global average/maximum pool operation on the channel to obtain feature mapping; this structure uses concat to fuse the features of the two processes, and the final feature mapping is obtained through the sigmoid process.

(2) $$M_{s}\left(F\right)=\sigma (f^{7\times 7}([\mathrm{Avgpool}\left(F\right);\mathrm{MLP}(\mathrm{Maxpool}(F)]))$$

**Figure 6:**
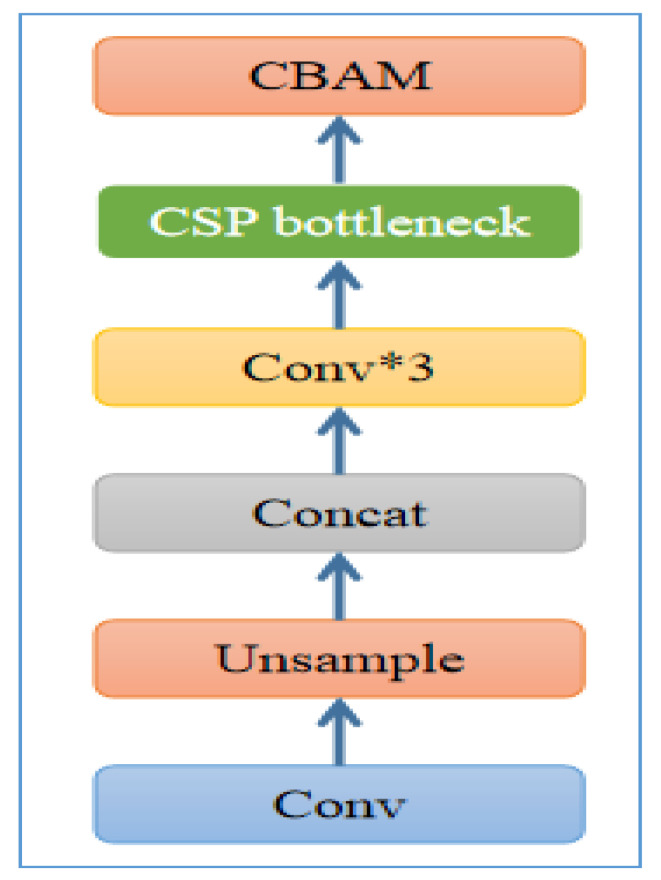
The CBAM model was added to each basic block, which integrates the output features immediately after the CSP bottleneck. Among them, Conv * 3 means that three convolution layers are superimposed together.

The insertion of CBAM in the model structure is shown in Figure 6, and we added CBAM to the feature pyramid before each extracted feature fusion to improve the model performance.

### 3.3. Adaptive Feature Fusion

**Figure 7:**
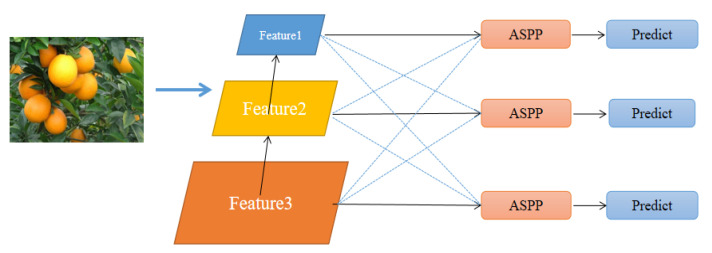
The feature map output from the model is fused adaptively.

Feature pyramid (FPN) is a standard structure for a target detection network to extract features with different resolutions. However, various scale features are processed separately without interaction, which limits the performance of the model. In the citrus dataset, there are many small target citrus fruits, which need to refine and reuse the underlying features. This paper used a fusion method for additional resolution features, called adaptive spatial feature fusion (ASFF). By learning to adaptively adjust the spatial weight of each scale feature during fusion, the scale invariance of the feature is improved. ASFF structure diagram is shown in Figure 7.

The formula of feature fusion of this method is shown in Equation (3). Where $x_{\mathrm{ij}}^{1}$ is the feature map of different feature layers, with three feature parameters α, β and γ. We used the three-layer features to multiply the weight parameters to obtain new fusion features. This structure has a good detection effect on small targets. We make full use of the fine-grained features in the underlying structure to identify small objects.

(3) $$y_{\mathrm{ij}}^{1}=\alpha_{\mathrm{ij}}^{1}\cdot x_{\mathrm{ij}}^{1}+\beta_{\mathrm{ij}}^{1}\cdot x_{\mathrm{ij}}^{2}+\gamma_{\mathrm{ij}}^{1}\cdot x_{\mathrm{ij}}^{3}$$

### 3.4. Model Pruning

Although the improved citrus detection network performs well in detection accuracy, it still has limitations when transplanted to Jetson nano edge computing equipment. To meet the diversity of different data distributions, the model will set many channels to extract rich features. However, the data features are relatively single for the citrus data set and do not need too many channels to fit the data. That is, there are many redundant parameters in the model, and not every channel in the characteristic diagram contains valuable information. In this section, we used the method of pruning the number of model channels to reduce the amount of calculation and parameters of the model, speed up the reasoning speed, and ensure the model’s accuracy through fine-tuning.

The batch normalization layer (BN) can forcibly pull back the deviation distribution in model training and then standardize it into the numerical level of normal distribution of mean and variance, which makes the activation function more sensitive and speeds up the training speed of the model. The calculation formula of BN layer is shown in Formulas (4) and (5). Activation size ${\hat{z}}_{out}$ and coefficient of each channel γ positive correlation, if γ too small, close to 0, the activation value is also very small.

(4) $${\hat{z}}_{i}=\frac{z_{in}-\mu_{B}}{\sqrt{\sigma_{B}^{2}+\epsilon }}$$

(5) $${\hat{z}}_{out}=\gamma {\hat{z}}_{i}+\beta$$

We used L2 regularization to constrain the parameters of the batch normalization layer for thinning and feature selection. After training a network normally, the coefficients of the BN layer are normally distributed, and adding L2 regularization to the loss function can make the weight value sparse and gradually approach 0. When the first training, redundant channels and features are selected by pruning, and relatively light feature weights are filtered and deleted. After convolution, the corresponding activation values of these channels are also relatively small. L2 regularization formula is shown in Equation (6).

(6) $$L=E_{\mathrm{in}}+\gamma \sum_{j}w_{j}^{2}$$

where L represents the sum of squares of weights added to the original loss and represents the square summation of each neuron in the vector weight, where E represents the training sample error without regularization, and w is the weight value in the model, which can be limited to 0, which can control the regularization trend. When the parameters increase, the model complexity will be constrained to a large extent. After pruning, the accuracy is restored by fine tuning. 

**Figure 8:**
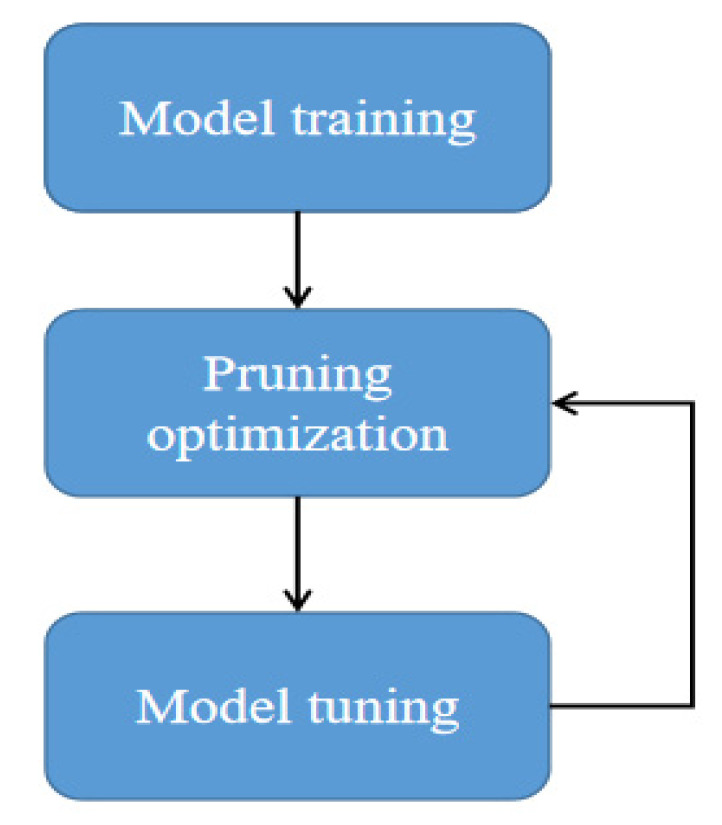
Model pruning process, to ensure accuracy, the model used in this paper is optimized twice.

The pruning process is shown in Figure 8. It is divided into three steps: training pruning fine-tuning. Firstly, the original model is used to train the citrus data set to obtain the weight; Taking the scaling factor in BN as a reference, L2 filters the weight value and deletes the number of channels whose weight is less than a certain threshold. Change the number of channels in the model network, make full use of the advantages of dense connection of convolution network, minimize the redundant channels and retrain.

## 4. Experiment and Results

In this part, firstly, we introduced the training details and methods used in this paper. Secondly, we designed ablation experiments and comparative experiments of different test sets on the improved model and evaluated on the edge computing equipment. Finally, we did a comparative experiment with the same advanced target detection algorithm. At the end of this section, we visualize the effect of citrus fruit recognition.

### 4.1. Experimental Training Setting

We used the transfer learning method to load the pre-train weight so that the model has stable parameters for training from the beginning, which speeds up the training speed and reduces the amount of data required for model fitting. The default input image resolution of the model is 608 pixel × 608 pixel × 3. Because there is only one category in the citrus dataset, the default feature dimension is 18. 

In training, we trained 50 epochs on the citrus data set, and set the constraint model using CIoU loss function and adam optimization algorithm β1 = 0.89, β2 = 0.99, ε = 109. Set batch size to eight. All training was conducted on NVIDIA RTX 2080 Ti graphics card. We used the operating system is Ubuntu 18.04, and the in-depth learning parallel acceleration Library of cuda10.2 and cudnn8.0 is used for acceleration training; PyTorch version is 1.7.

### 4.2. Ablation Experiment

We used the average accuracy, the detection speed of a single picture in Jetson nano, and the recall as the evaluation indexes. Formulas (7)–(9) is the calculation method of the average accuracy. Among them, average accuracy is a popular evaluation method of the target detection model, which is used to evaluate whether the model detects objects in the image accurately.

(7) $$P=\frac{TP}{TP+FP}\times 100\%$$

(8) $$R=\frac{TP}{TP+FN}\times 100\%$$

(9) $$R=\frac{TP}{TP+FN}\times 100\%$$

where *P* is the correct rate (%), *R* is the recall rate (%), *TP* is the real-positive sample, *FP* is the false-positive sample, and *FN* is the false-negative sample.

To understand the model performance and prove the effectiveness of the optimized model in this paper, we compared each improved part and conducted the ablation experiment of the model on all citrus test set, as shown in Table 1. 

It can be seen from the table that after adding the CBAM module, the detection accuracy of the model is improved by 2.39%, but the detection time is increased by 40 ms. After adding ASFF, the detection accuracy is improved by 0.44%. To recover and speed up the reasoning speed of the model, the detection speed of citrus by the pruned model is 180 ms/frame.

In this paper, to ensure the accuracy of the model, we use twice pruning to optimize the model, compared the results of pruning processes, as shown in the Table 2. After the first pruning, the memory occupied by the model is reduced by 6 M when a small amount of accuracy is lost. After the second pruning, the memory volume of the model is only 21 M, while ensuring accuracy, we reduced the amount of calculation and parameters of the model, which provides convenience for deployment to edge computing devices.

### 4.3. Comparative Test of Different Occlusion Degrees

In this section, we used the improved model to conduct comparative experiments on test sets A and B, respectively, among them, dataset A is the slightly occluded citrus fruit image, and dataset B is the heavily occluded citrus fruit image, the results as shown in Table 3. The recognition accuracy of the improved model for slightly occluded citrus fruit images is 96.01%, which is higher than that of the basic model by 0.57%, and the recognition accuracy of heavily occluded citrus fruit images is 89.41%, which is higher than that of the basic model by 2.55%. Experiments show that the model can improve the detection effect of citrus fruits under different occlusion degrees, which proves the effectiveness of the method proposed in this paper.

### 4.4. Comparative Experiment of Different Target Detection Models

We have carried out comparative experiments with the same advanced target detection models, including FCOS, YOLOv4, and YOLOv3, as shown in Table 4. The accuracy of our improved model is higher than that of other baselines. Similarly, the detection speed in 2080Ti is 83 FPS/s, which is increased by 10 and 14 FPS/s, respectively, compared with YOLOv4 and YOLOv3.

**Figure 9:**
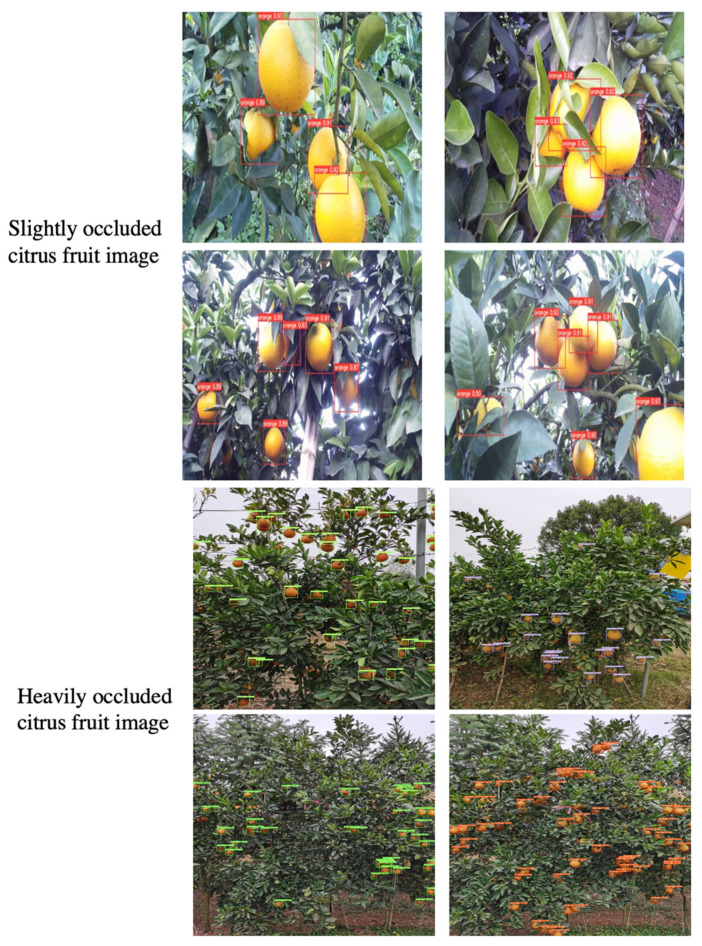
We visualized the detection effect of citrus fruits under different occlusion levels, In the figure, the words above all detected citrus fruits are "orange" and their confidence.

Figure 9 visualizes the effect of our citrus detection model. We showed the detection effect of citrus fruits with mild occlusion and severe occlusion, respectively.

## 5. Conclusions

We designed a real-time citrus fruit detection system combining a mobile operation platform and edge computing equipment to solve the problems of inconvenient data capture in mountain orchards and the unbalanced speed and accuracy of the target detection model at the edge. By expanding the current most advanced target detection network and improving its feature extraction and reasoning ability, the citrus fruit image data collected by UAV is detected on the edge computing equipment. The test results show that when there are targets with different degrees of occlusion in the natural orchard environment, the accuracy and detection speed of the model are up, which is improved compared with the original baseline. Specifically, we carry out real-time target detection at the edge in the currently popular UAV captured image scene, which is suitable for working in different environments. The main contributions of this paper are as follows:

1. Benefiting from the strong maneuverability and high security of UAV aerial images, and not limited to the actual scene of Mountain Orchard, we collected and labeled 1800 citrus images for target detection model training.
2. Improve the current most advanced target detection model and use CBAM attention mechanism and data enhancement to improve the generalization and accuracy of the model; At the same time, the L2 regularization method constraint model adds to delete the redundant channel with the minimum weight of 30% and fine-tune it. It is detected in the Jetson nano edge computing device. The results are achieved. While ensuring accuracy, a faster detection speed is achieved.

In this paper, for citrus detects by combining visual algorithm, mobile flight equipment, and edge operation platform, we hope this work can provide some help for fruit farmers to estimate yield and improve the operation efficiency of mountain orchards.

## 6. Discussion and Future Work

It is hard to say that our approach has no drawbacks. We mainly focus on ripe citrus fruits and provide technical support for yield estimation. Still, it can also lead to errors when identifying many immature citrus fruits, turning green leaves into natural citrus. To overcome this problem, we will collect images of citrus fruits with different maturity levels for analysis. In addition, the use of UAV technology equipment in mountain orchards often requires professional control. If the shooting distance is close, the UAV equipment can easily touch the branches, causing the operation to fail. In future work, we will also use UAVs to collect and experiment with citrus fruit images of different heights.

Despite these problems, we are committed to using smart devices such as sensors to reduce human resource consumption. We can purchase the edge computing devices used in this article for only 800 RMB. At the same time, compared with the ground imaging system, UAV can collect fruit data from different parts of citrus tree in an all-round way without the influence of orchard location, geographical conditions, and climatic environment, which can reduce the labor time and operation costs of fruit growers. The results show that our recognition of citrus fruits is still effective.

## Figures and Tables

**Table 1:** Model Ablation Experiment.

Model	AP/%	Speed/ms	Recall/%
YOLOv5s	91.03	270	87.13
YOLOv5s + CBAM	93.42	310	88.21
YOLOv5s + CBAM + ASFF	93.86	320	88.91
YOLOv5s + CBAM + ASFF + Purning	93.32	180	88.78

**Table 2:** Model results of twice pruning.

Model	Model Size/MB	AP/%
Original	33	93.86
First	27	93.55
Second	21	93.32

**Table 3:** Comparison of citrus fruit data sets with different occlusion degrees.

Model	Dataset. A (AP/%)	Dataset. B (AP/%)
Original	95.44	87.86
Ours	96.01	90.41

**Table 4:** Model comparison experiment.

Model	AP/%	FPS (In 2080ti)/s
FCOS	90.76	47
YOLOv3	91.21	69
YOLOv4	91.97	73
Ours	93.32	83

## Data Availability

Not applicable.
